# Schlafen 12 Is Prognostically Favorable and Reduces C-Myc and Proliferation in Lung Adenocarcinoma but Not in Lung Squamous Cell Carcinoma

**DOI:** 10.3390/cancers12102738

**Published:** 2020-09-24

**Authors:** Sarmad Al-Marsoummi, Jonathan Pacella, Kaylee Dockter, Matthew Soderberg, Sandeep K. Singhal, Emilie E. Vomhof-DeKrey, Marc D. Basson

**Affiliations:** 1 Department of Biomedical Sciences, School of Medicine and the Health Sciences, University of North Dakota, Grand Forks, ND 58202, USA; sarmad.al.marsoummi@ndus.edu (S.A.-M.); jonathan.pacella@und.edu (J.P.); kaylee.dockter@und.edu (K.D.); matthew.soderberg@und.edu (M.S.); emilie.dekrey@med.und.edu (E.E.V.-D.); 2 Department of Pathology, School of Medicine and the Health Sciences, University of North Dakota, Grand Forks, ND 58202, USA; sandeep.singhal@und.edu; 3 Department of Surgery, School of Medicine and the Health Sciences, University of North Dakota, Grand Forks, ND 58202, USA

**Keywords:** Schlafen 12, c-myc, lung adenocarcinoma, lung squamous cell carcinoma, non-small cell lung cancer

## Abstract

**Simple Summary:**

Different subtypes of lung cancer respond differently to treatment. To understand the signaling pathways that dictate the aggressive behavior of lung adenocarcinoma vs. lung squamous cell carcinoma, we performed a survival analysis that demonstrated that the protein Schlafen 12 correlates with better survival in patients with lung adenocarcinoma but not patients with lung squamous cell carcinoma, indicating specificity of the effect of the SLFN12 pathway in lung cancer subtypes. We investigated this specific effect by confirming the ability of Schlafen 12 to reduce cell proliferation of lung adenocarcinoma cells in vitro but not that of lung squamous cell carcinoma. Moreover, we demonstrated that Schlafen 12 acts through translational inhibition of c-myc protein in lung adenocarcinoma cells. The ability to delineate the exact signaling pathways that modulate each lung cancer subtype’s aggressive behavior will help in the future development of precision medicine to target this challenging disease.

**Abstract:**

Schlafen 12 (SLFN12) is an intermediate human Schlafen that induces differentiation in enterocytes, prostate, and breast cancer. We hypothesized that SLFN12 influences lung cancer biology. We investigated survival differences in high versus low SLFN12-expressing tumors in two databases. We then adenovirally overexpressed SLFN12 (AdSLFN12) in HCC827, H23, and H1975 cells to model lung adenocarcinoma (LUAD), and in H2170 and HTB-182 cells representing lung squamous cell carcinoma (LUSC). We analyzed proliferation using a colorimetric assay, mRNA expression by RT-qPCR, and protein by Western blot. To further explore the functional relevance of SLFN12, we correlated SLFN12 with seventeen functional oncogenic gene signatures in human tumors. Low tumoral SLFN12 expression predicted worse survival in LUAD patients, but not in LUSC. AdSLFN12 modulated expression of SCGB1A1, SFTPC, HOPX, CK-5, CDH1, and P63 in a complex fashion in these cells. AdSLFN12 reduced proliferation in all LUAD cell lines, but not in LUSC cells. SLFN12 expression inversely correlated with expression of a myc-associated gene signature in LUAD, but not LUSC tumors. SLFN12 overexpression reduced c-myc protein in LUAD cell lines but not in LUSC, by inhibiting c-myc translation. Our results suggest SLFN12 improves prognosis in LUAD in part via a c-myc-dependent slowing of proliferation.

## 1. Introduction

Lung cancer is the leading cause of human cancer death, having caused 1.76 million deaths worldwide in 2018 [1]. Surgery, cytotoxic chemotherapy, and radiotherapy remain the mainstay of lung cancer therapy, as no more specifically targeted therapy is available [2]. Lung cancer is classified into the rare entity of small cell lung cancer and the more common non-small cell lung cancer (NSCLC) [3]. Histologically, NSCLC is divided into lung adenocarcinoma (LUAD), which comprises 50% of the cases, and lung squamous cell carcinoma (LUSC), which makes up 40% of the cases, with an additional 10% that are classified as large cell undifferentiated carcinoma [4]. Intricate signaling molecular pathways fuel the aggressive behavior and heterogeneity of each histological subtype of NSCLC. Therefore, it is imperative to understand the disparate signaling pathways that modulate the aggressiveness of each subtype of lung cancer to design better-targeted precision therapy.

The Schlafens are a group of diverse genes that are expressed in humans as well as rodents. Schlafens are classified into short, intermediate, and long families based on their size and structure. While rodents express all three families of Schlafens, humans express four long Schlafens (hSLFN5, 11, 13, and 14), only one intermediate Schlafen (hSLFN12), and no short Schlafens.

The long Schlafens have recently been implicated in lung cancer biology in a complex fashion. SLFN5 expression has been reported to be prognostically favorable in lung cancers [5], but it activates epithelial to mesenchymal transition in vitro [6]. High SLFN11 expression is also associated with a favorable outcome in lung cancers, perhaps at least in part because it sensitizes lung cancer to specific cytotoxic drugs such as DNA alkylating agents and PARP inhibitors [7,8,9]. Still, how these long Schlafens affect lung cancer has not been stratified between adenocarcinoma and squamous cell carcinoma of the lung. In addition, long Schlafens have both nuclear targeting sequences and DNA binding ability [10], while the intermediate SLFN12 lacks such a sequence and is localized to the cytoplasm. SLFN12 would thus not be expected to act in the same fashion as a long Schlafen. Our previously published work identified SLFN12 as modulating differentiation in intestinal epithelium, breast cancer, and prostate cancer [11,12,13]. We now want to understand whether SLFN12 plays a major role in lung cancer biology and prognosis and whether this role might differ between adenocarcinoma and squamous carcinoma of the lung.

Our analysis demonstrated that high versus low SLFN12 expression is able to classify the lung adenocarcinoma, but not squamous cell carcinoma patients into good versus poor survival. SLFN12 reduced the proliferation of LUAD cell lines, but not LUSC cell lines in vitro. Genomic analysis of human lung cancers suggested that SLFN12 expression inversely correlated with c-myc gene signature in LUAD, but not in LUSC, and we validated this finding at the protein level using cell lines. Furthermore, we demonstrated SLFN12 mechanistically acts through inhibition of c-myc protein translation. In conclusion, our results indicate that SLFN12 plays a favorable prognostic role in lung adenocarcinoma at least in part by modulating c-myc expression. This pathway may be a target for future precision-targeted therapy for lung adenocarcinoma.

## 2. Results

### 2.1. Schlafen12 Expression Correlates with Better Survival in Lung Adenocarcinoma

**Figure 1:**
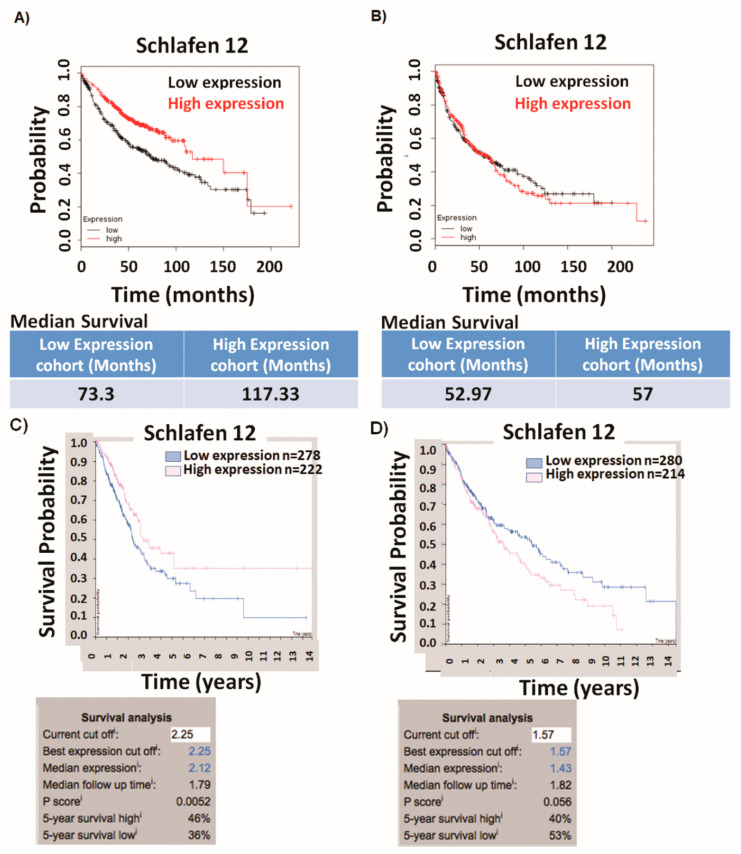
Schlafen12 (SLFN12) correlates with survival of lung adenocarcinoma. Survival analysis of publicly available dataset of cohorts of patients with lung cancer (http://kmplot.com/analysis/) shows that (**A**) SLFN12 expression correlates with overall survival after diagnosis in lung adenocarcinoma (LUAD) (*n* = 719, *p* < 0.01) and (**B**) SLFN12 expression does not correlate with overall survival after diagnosis in patients with lung squamous cell carcinoma (LUSC) (*n* = 524, *p* = 0.78). The median expression of SLFN12 was used as a cutoff value and median survival in months was calculated for both high and low expression cohort. Parallel survival analysis from a different tool (http://www.proteinatlas.org) confirms that (**C**) SLFN12 mRNA expression correlates with overall survival after diagnosis in LUAD (*n* = 500, *p* = 0.0052), while (**D**) SLFN12 mRNA expression does not correlate with overall survival in patients with LUSC (*n* = 494, *p* = 0.0056). fragments per kilobase of exon model per million reads mapped (FPKM) value of SLFN12 gene that yielded the maximum survival difference was used as a cutoff to separate the two cohorts.

Our analysis using the publicly available km-plotter tool [14] of gene expression profiles in a cohort of patients with non-small cell lung cancer showed that higher SLFN12 expression correlated with better survival in patients with lung adenocarcinoma (hazard ratio = 0.59, *n* = 719, *p*-value < 0.0001). The median survival for patients with adenocarcinoma who expressed high levels of SLFN12 was 117.3 months versus 73.3 months in patients with low SLFN12 expression (Figure 1A). In contrast, SLFN12 expression did not correlate with survival in patients with lung squamous cell carcinoma subtype (hazard ratio = 1.03, *n* = 524, *p*-value = 0.78) (Figure 1B) [15]. We validated these findings by analysis using a different online tool from the human protein atlas [16,17], which shows similar results (Figure 1C,D).

### 2.2. Schlafen12 Changed the Differentiation Markers and Reduced Proliferation in Lung Adenocarcinoma Cells

Because SLFN12 has been implicated in the regulation of differentiation in other epithelial tissues, we next sought to examine the effect of exogenous SLFN12 overexpression on a set of differentiation markers in a panel of lung adenocarcinoma and squamous cell carcinoma cell lines.

**Figure 2:**
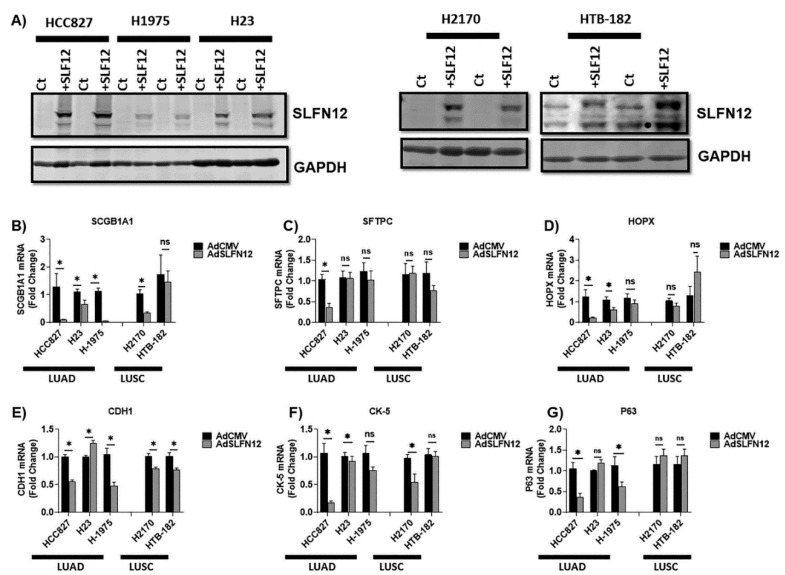
SLFN12 modulates mRNA levels of differentiation markers in lung cancer cells. (**A**) Representative Western blot images confirm successful SLFN12 overexpression in lung adenocarcinoma cells (HCC827, H1975, and H23 cells) and in lung squamous cell carcinoma cells (H2170 and HTB-182 cells). Glyceraldehyde 3-phosphate dehydrogenase (GAPDH) was used as a housekeeping protein control. (ct = background adenovirus AdCMV, SLF12 = AdSLFN12). mRNA analysis by Primer-probe qPCR, 72 hours after AdSLFN12 or AdCMV treatment for the following: (**B**) SCGB1A1, (**C**) SFTPC, (**D**) HOPX, (**E**) CDH1, (**F**) CK-5, and (**G**) P63 in HCC827, H23, H1975, H2170, and HTB-182 cells (*n* = 3–12) (Hypoxanthine-guanine phosphoribosyltransferase (HPRT) was used as a reference gene, data normalized to AdCMV group, ns = non-significant, * *p* < 0.05). All data are represented as mean ± SEM. Detailed information about western blot can be found at Figure S1.

SLFN12 overexpression using the adenoviral vector AdSLFN12 was confirmed by Western blot (Figure 2A). Overexpression of SLFN12 significantly reduced mRNA levels of the adenocarcinoma differentiation marker SCGB1A1 in all of the LUAD cells studied (HCC827, H23, and H1075) and in one LUSC cell line (H2170 cells) compared with treatment with AdCMV as a control. The expression of a second adenocarcinoma differentiation marker, SFTPC, was significantly reduced by AdSLFN12 treatment in only one LUAD cell (HCC827), while AdSLFN12 significantly reduced HOPX mRNA levels in two LUAD cells (HCC827 and H23) with no significant changes in LUSC cells (Figure 2B–D).

We next examined the effects of AdSLFN12 on two common markers of squamous cell differentiation. AdSLFN12 significantly reduced the expression of the squamous cell marker P63 in two LUAD cell lines (HCC827 and H1975), with no significant changes in LUSC cells, while the mRNA level of the squamous cell marker CK5 was reduced significantly after AdSLFN12 treatment in two LUAD cell lines (HCC827 and H23 cells) and only one LUSC cell line (H2170 cells) (Figure 2E–G). Only one LUAD cell line (H23 cells) showed a significant increase in expression of the epithelial marker CDH1 after AdSLFN12 treatment. Moreover, we performed RT-qPCR using multiple housekeeping genes (RPLP0, ACTB, B2M, POL2A), which validated HPRT as stable housekeeping gene (Figure S2). Moreover, time-course analysis of mRNA levels at 48 and 72 hours after AdSLFN12 transfection demonstrated a similar trend in mRNA levels of the differentiation markers as seen previously (Figure S3).

**Figure 3:**
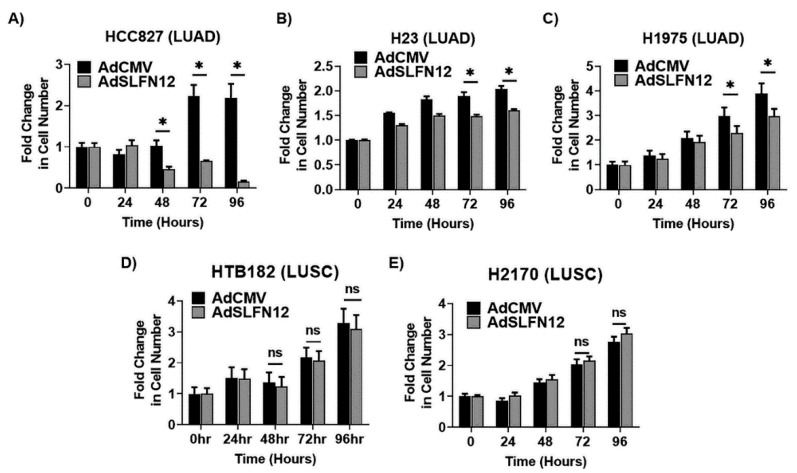
Schlafen 12 reduces cell number in lung adenocarcinoma cells, but not in lung squamous cell carcinoma cells. In lung adenocarcinoma, Schlafen12 overexpression using an adenoviral vector (AdSLFN12) significantly reduced cell number at 72 and 96 hours after AdSLFN12 treatment compared with the empty vector (AdCMV) treatment in the following: (**A**) HCC827, (**B**) H23, and (**C**) H1975 cells (*n* = 9–12, * *p* < 0.05). Data represent mean fold change in cell number normalized to cell number at zero hours. In contrast, in lung squamous cell carcinoma, AdSLFN12 treatment did not reduce the cell number in (**D**) HTB-182 cells (*n* = 9, ns = non-significant) or (**E**) H2170 cells (*n* = 9, ns = non-significant), compared with AdCMV treatment. Data represent fold change in cell number normalized to zero hours (* *p* < 0.05). All data are represented as mean ± SEM.

Because the effects of SLFN12 on these differentiation markers were inconsistent among cell lines and difficult to interpret, we next examined the effect of SLFN12 overexpression on proliferation. Exogenous SLFN12 expression using AdSLFN12 significantly reduced cell numbers of HCC827 cells at 48 hours compared with cells infected with AdCMV control (0.67-fold ± 0.01 vs. 2.23-fold ± 0.26, *n* = 10, *p* < 0.01, Figure 3A). This anti-proliferative effect was reproduced in two additional lung adenocarcinoma cell lines. In H23 cells, AdSLFN12 transfection reduced cell number starting at 72 hours compared with AdCMV control (1.50-fold ± 0.03 vs. 1.82-fold ± 0.06, *n* = 10, *p* < 0.01, Figure 3B). In H1975 cells, cell number was reduced at 72 hours after AdSLFN12 transfection compared with cells infected with AdCMV control (2.96-fold ± 0.30 vs. 3.89-fold ± 0.41, *n* = 10, *p* < 0.01, Figure 3C).

Flow cytometry analysis demonstrated SLFN12 overexpression using AdSLFN12 significantly increased apoptotic cells in LUAD cells (H23, HCC827, and H1975 cells) compared with the control cells transfected with AdCMV (Figure S4).

Additionally, flow cytometric analysis of cell proliferation using Tag-it proliferation dye demonstrated that overexpressing SLFN12 using an AdSLFN12 reduced the proliferation in all three LUAD cells (H23, HCC827, and H1975 cells) compared with the control cells transfected with AdCMV. This further confirms the antiproliferative effect of SLFN12 (Figure S5). Moreover, AdSLFN12 transfection significantly increased the proportion of cells arrested in the G0/G1-phase compared with AdCMV transfection in HCC827 cells (68.30% ± 1.19% vs. 50.52% ± 2.93%, *n* = 5, *p* < 0.05), H23 cells (62.28% ± 0.16% vs. 59.60% ± 0.29%, *n* = 6, *p* < 0.05), and H1975 cells (46.43% ± 3.83% vs. 24.50% ± 0.60%, *n* = 3, *p* < 0.05), (Figure S6).

In contrast, AdSLFN12 infection did not alter cell number in either of the two squamous cancer cell lines. Cell number of AdSLFN12-infected HTB-182 cells even 96 hours after infection resembled that of AdCMV-transfected control cells (3.10-fold ± 0.44 vs. 3.29-fold ± 0.45, *n* = 9, *p* = 0.76, Figure 3D). Similarly, AdSLFN12-transfected H2170 cell number was similar to that of AdCMV-infected control cells at 96 hours (3.03-fold ± 0.06 vs. 2.77-fold ± 0.05, *n* = 10, *p* = 0.38, Figure 3E).

### 2.3. SLFN12 Correlated with the MYC Downstream Gene Signature in Lung Adenocarcinoma Differently Than Lung Squamous Cell Carcinoma

**Figure 4:**
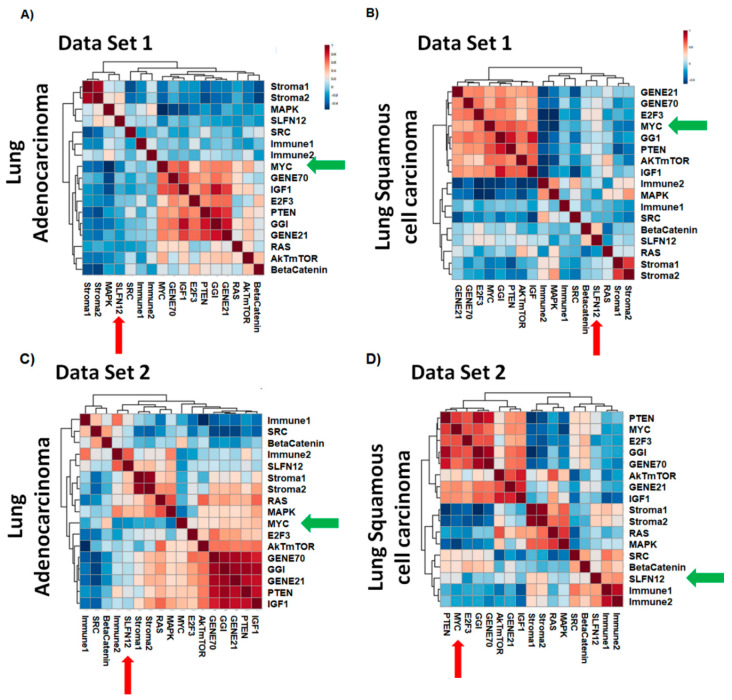
SLFN12 correlates with gene signatures in human lung carcinoma. Heatmap of correlation levels between 17 previously defined cancer-related gene signatures using two datasets of lung cancer patients. Heat maps derived from dataset 1 are shown in (**A**) for lung adenocarcinoma (*n* = 77) and in (**B**) for lung squamous cell carcinoma (*n* = 73). Heat maps derived from dataset 2 are shown for (**C**) lung adenocarcinoma (*n* = 40) and (**D**) lung squamous cell carcinoma (*n* = 18).

To investigate the possible mechanism of SLFN12 effect on cell proliferation in LUAD, gene signature expression analysis was performed on two lung cancer datasets (dataset 1 and dataset 2), examining seventeen gene modules from our previously published and defined signatures that describe pathways critical for oncogenic processes [18]. Four gene modules appeared differentially correlated with SLFN12 depending upon the histological subtype of the tumor. These included gene signatures downstream and associated with MAPK, beta-catenin, Akt-mTOR, and myc. In particular, in each dataset, the correlation between SLFN12 and the myc-associated gene signature differed between the adenocarcinoma (Figure 4A,C) and squamous cell lung cancer subtypes (Figure 4B,D). Although the correlation levels did not achieve statistical significance, perhaps because of the relatively small sample size, we observed a trend toward negative correlation in adenocarcinoma in each data set (Pearson correlation, dataset 1 = −0.264, dataset 2 = −0.3196) (Figure 4A,C), while we observed weak positive correlations between SLFN12 and MYC in squamous cell carcinoma in both data sets (Pearson correlation, data set 1 = 0.19668, data set 2 = 0.050284) (Figure 4B,D). The full list of genes that correlated with SLFN12 in lung adenocarcinoma vs. lung squamous cell carcinoma can be seen in supplemental file (Tables S1–S3).

Moreover, bioinformatics analysis of datasets [19,20] in lung adenocarcinoma identifies the correlation of SLFN12 with other known lung adenocarcinoma markers like KRT7, Napsin A, and TTF-1. SLFN12 negatively correlated with KRT-7 and positively correlated with Napsin-A (Figure S7A). As the correlation of SLFN12 with TTF-1, an important diagnostic marker for lung adenocarcinoma, showed inconsistent results using the two available probes for RNAseq analysis, we further performed RT-qPCR in the three LUAD cell line models studied here to analyze mRNA levels of TTF-1. Our RT-qPCR results show that overexpression of SLFN12 significantly increased TTF-1 mRNA levels in two of the three lung adenocarcinoma cells (HCC827 and H1975) (Figure S7B).

### 2.4. Schlafen12 Overexpression Reduced C-Myc Protein Levels in Lung Adenocarcinoma, but Not Lung Squamous Cell Carcinoma

To investigate the possible mechanism of SLFN12 effect on cell proliferation of LUAD cells, we analyzed the effects of SLFN12 on c-myc in the cell lines under study. The myc-associated signature was singled out for further study because of the importance of the myc pathway in cell proliferation and because the correlation of myc-associated genes with SLFN12 was different between LUAD and LUSC. In particular, we isolated c-myc for further investigation because of the importance of c-myc in cell proliferation in general and in lung cancer specifically [21].

**Figure 5:**
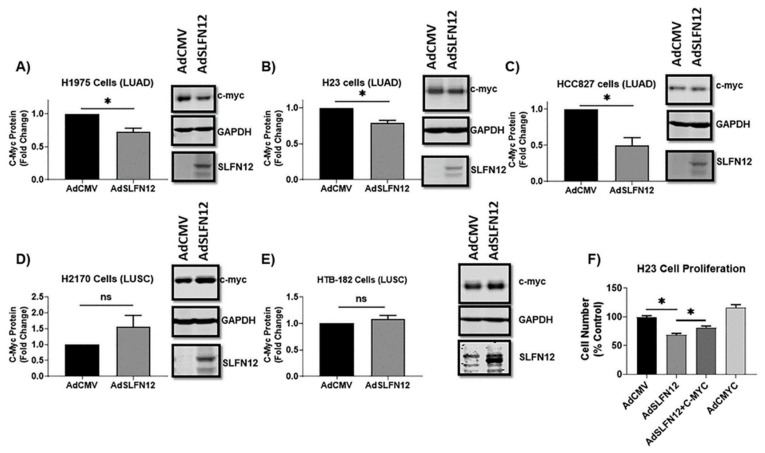
Schlafen12 reduces c-myc protein levels, causing reduced proliferation. Protein levels of c-myc analyzed by Western blot in (**A**) H1975 (*n* = 3), (**B**) H23 (*n* = 3), (**C**) HCC827 (*n* = 6), (**D**) H2170 (*n* = 3), and (**E**) HTB-182 (*n* = 3) cells with representative Western blot images, after treatment with AdSLFN12 or AdCMV (ns = non-significant, * *p* < 0.05). GAPDH was used as a reference protein. All data represent analysis performed 72 hours after AdSLFN12 treatment, except that H23 cells were analyzed 48 hours after AdSLFN12 treatment because the substantial reduction in cell number by 72 hours impeded protein isolation at this later time point. Lower blot images in each panel represent SLFN12 labeling to confirm successful SLFN12 overexpression at the protein level. (**F**) Cell proliferation assessed by toluidine blue staining of H23 cells 72 hours after infection with adenovirus expressing SLFN12 (AdSLFN12), c-myc (AdCMYC), or background adenovirus (AdCMV) as control; data show co-expression of c-myc (AdCMYC) with SLFN12 (AdSLFN12) significantly attenuated the anti-proliferative effect of AdSLFN12 (one-way analysis of variance (ANOVA), * *p* < 0.05). All data are represented as mean ± SEM. Detailed information about western blot can be found at Figure S1.

AdSLFN12 significantly reduced c-myc protein levels in LUAD cell lines. SLFN12-infected HCC827 cells displayed a 27.3% ± 5.6% reduction (*n* = 6, *p* < 0.05) at 72 hours after infection versus empty vector controls (Figure 5A), while H23 cells displayed a 21.0% ± 3.5% reduction (*n* = 3, *p* < 0.05) at 48 hours (Figure 5B) and H1975 cells displayed a 50.0% ± 10.6% reduction (*n* = 6, *p* < 0.05) at 72 hours (Figure 5C).

Knockdown of SLFN12 using an adenoviral vector expressing short hairpin RNA directed against SLFN12 (ShRNA-SLFN12) did not significantly change c-myc protein levels in H23, Hcc827, and H1975 LUDA cells compared with adenoviral vector expressing short hairpin non-targeted RNA sequence(shRNA-Cont) as control (Figure S8).

In contrast, lung squamous H2170 cells had a c-myc protein expression that was 155.6% ± 35.4% of empty-vector-transfected control values 72 hours after AdSLFN12 transfection (Figure 5D), while in HTB-182 cells, c-myc was 107.9% ± 7.2% of control values 72 hours after AdSLFN12 infection (*n* = 3, *p* = n.s. for each) (Figure 5E).

To establish that the ability of SLFN12 to reduce cell number in LUAD is at least in part through its effect in reducing c-myc protein levels, we analyzed cell numbers using toluidine blue cell staining after co-expression of both SLFN12 and c-myc using adenoviral vectors in H23 lung adenocarcinoma cells (we used H23 LUAD cells for this experiment because this cell line showed the least apoptotic cell death, as shown previously). C-myc co-expression with SLFN12 in H23 cells significantly attenuated the ability of SLFN12 to reduce cell number in H23 cells; AdSLFN12 transfection alone reduced the H23 cell numbers to 68.84% ± 2.41% versus 81.39% ± 2.76% in cells transfected with both AdSLFN12 + AdC-MYC (Figure 5F).

### 2.5. Schlafen12 Reduced C-Myc Protein Translation in H23 Lung Adenocarcinoma Cells

**Figure 6:**
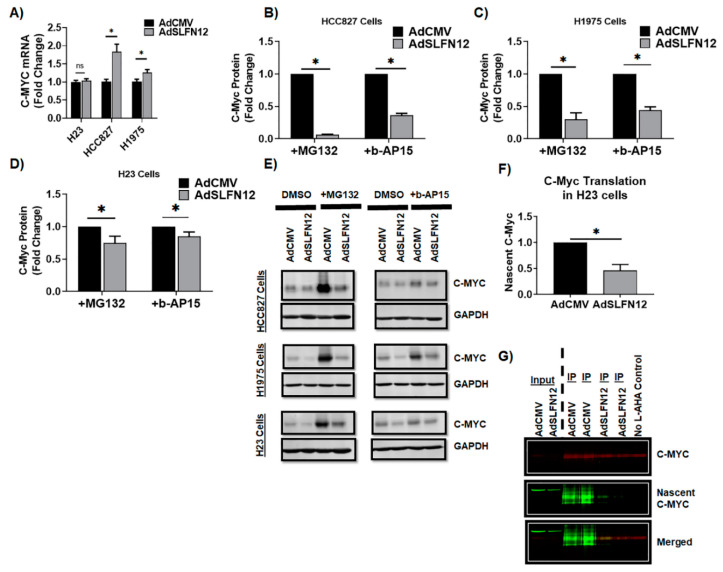
Schlafen 12 reduces c-myc protein translation. (**A**) C-myc mRNA levels analyzed by primer-probe RT-qPCR in HCC827 (*n* = 6), H23 (*n* = 6), and H-1975 (*n* = 6) LUAD cells, 72 hours after AdSLFN12 or AdCMV treatment. HPRT was used as a reference gene and data were normalized to the AdCMV controls (* *p* < 0.05, ns = non-significant). C-myc protein levels analyzed by Western blot in (**B**) H1975 (*n* = 3), (**C**) H23 (*n* = 3), and (**D**) HCC827 (*n* = 6) cells, with representative Western blot images shown in (**E**), 72 hours after infection with AdSLFN12 or AdCMV control, in the presence of 10 µM of the proteasomal inhibitor MG132 or 1 µM of the deubiquitylase inhibitor b-AP15 (* *p* < 0.05, GAPDH used as a reference protein). (**F**,**G**) C-myc protein translation in H23 cells (*n* = 3), 68 hours after treatment withAdSLFN12 or AdCMV control, performed by Click-iT reaction of L-AHA metabolic labeling of newly synthesized (nascent) c-myc protein (green), after immunoprecipitating total c-myc protein (red). Nascent c-myc protein (green) was normalized to the total immunoprecipitated c-myc (red) and all data were then normalized to the control AdCMV values (* *p* < 0.05). All data are represented as mean ± SEM. Detailed information about western blot can be found at Figure S1.

We next sought to probe the mechanism by which SLFN12 regulates c-myc levels. Our mRNA level analysis excluded transcriptional inhibition of c-myc by SLFN12 (Figure 6A), as SLFN12 did not decrease c-myc mRNA, and indeed seemed to increase it in two of the three cell lines. Next, because SLFN12 regulates ZEB1 in breast cancer through translational inhibition [12], and CDX2 in intestinal epithelial cells by modulating the deubiquitylases USP14 and UCHL5 [11], we decided to separately inhibit the proteasome and UCHL5/USP14 deubiquitylase activity to determine if either would block the ability of SLFN12 to reduce c-myc protein. However, neither proteasomal inhibition nor inhibition of these deubiquitylases blocked SLFN12 regulation of c-myc (Figure 6B–E).

In light of these results, we next turned our attention to the possibility that SLFN12 might modulate c-myc protein translation. Indeed, analysis of c-myc protein translation using L-AHA metabolic labeling and Click-iT chemistry demonstrated that SLFN12 overexpression through AdSLFN12 transfection of H23 cells significantly reduced c-myc translation by 55% compared with control cells transfected with the empty vector control AdCMV (Figure 6F,G).

## 3. Discussion

Non-small cell lung cancer is classified into adenocarcinoma (LUAD) and squamous cell carcinoma (LUSC) [3]. This study identified SLFN12 as prognostically favorable in LUAD, but not in LUSC. Although SLFN12 has previously been shown to regulate differentiation in other epithelial cell types [11,12,13], SLFN12 changed mRNA levels of differentiation markers (SCGB1A1, SFTPC, HOPX, CDH1, CK-5, and P63) in a complex and inconsistent fashion among LUAD and LUSC cell lines. However, SLFN12 tended to correlate differently with myc-associated gene signatures between human lung adenocarcinomas and human lung squamous cell carcinomas, while SLFN12 reduced both c-myc protein levels and cell proliferation only in lung adenocarcinoma cell lines, with no similar effects in LUSC. SLFN12 acted in LUAD cells by reducing the translation of c-myc protein.

The Schlafens are a group of genes that are expressed in diverse species, including humans and rodents. Schlafens are classified based on their size and structure into short, intermediate, and long families. However, humans express only long (SLFN5, SLFN11, SLFN12, SLFN13, SLFN14) Schlafens and one intermediate Schlafen, which is SLFN12. Schlafens play diverse roles in cell differentiation, immune cell proliferation and maturation, and cancer [22,23]. The role of Schlafens in lung cancer is poorly understood. High expression of either SLFN5 or SLFN11 has been reported to be prognostically favorable for lung cancer [24,25], and SLFN11 in particular has also been reported to sensitize lung cancer cells to DNA alkylating agents and poly ADP ribose polymerase (PARP) inhibitors [8,24]. However, SLFN5 has actually been found to activate the epithelial–mesenchymal transition (EMT) axis in A549 cells, which might be expected to enhance metastatic potential [6]. Furthermore, SLFN11 and SLFN5 are long Schlafens that possess a nuclear import signal and a putative RNA helicase domain, and bind directly to chromosomal DNA. In contrast, the intermediate SLFN12 lacks such sequences and is localized to the cytoplasm [11,26]. Therefore, one would not necessarily expect SLFN12 to act in the same fashion as the long Schlafen proteins. SLFN12 induces differentiation in the enterocyte, in prostate cancer, and in breast cancer [12,13,27]. It appears to act in the cytosol by modulating the translation and degradation of transcription factors that in turn alter cell phenotype [11,12]. SLFN12 also sensitizes cancer cells, including lung cancer, to PDE3A inhibitors [28,29]. Our analysis identified SLFN12 as a favorable prognostic factor in lung cancer, but more importantly, stratified this survival correlation by histological subtype. The prognostic implications of SLFN12 expression appear exclusive to lung adenocarcinoma and do not apply to squamous cell carcinoma. Although the KM plotter data and the Cancer Genome Atlas data are not completely independent, we used both to study the effects of SLFN12 on human lung cancer biology. Analysis of the metadata from these datasets suggests that there is only an 8% overlap in lung cancer samples between these two datasets. Moreover, the KM Plotter data were generated using the Affymetrix HT Human Genome U133A Array [9], whereas the Human Protein Atlas transcript profiling was generated by mRNA sequencing (RNA-Seq), performed on Illumina HiSeq2000/2500 machines (Illumina, San Diego, CA, USA), using the standard RNA-Seq protocol with a read length of 2 × 100 bases [10]. Such different methodologies do not uncommonly yield different results. Thus, obtaining similar results implicating SLFN12 in the prognosis of human lung adenocarcinoma from both datasets validates and adds to the rigor of our conclusion that SLFN12 is prognostically important for these tumors.

Our results not only indicate the prognostic significance of SLFN12 in LUAD, but also suggest that SLFN12 and its downstream effectors may be useful targets for the future design of precision medicine for adenocarcinoma of the lung. Although we focused here on adenocarcinoma and squamous cell carcinoma of the lung, it would be interesting to examine the effect of SLFN12 on large cell and small cell lung cancer survival, but the databases available to us did not contain sufficient sample size to perform such analyses.

As we knew that SLFN12 alters cell differentiation, we initially sought an explanation for the apparent protective implications of high SLFN12 expression in lung adenocarcinoma by examining the expression of several important differentiation markers. CK-5 and P63 were picked because they are both markers of squamous cell carcinoma [3,30], and SFTPC, SCGB1A1, and HOPX are markers of LUAD [31], while CDH1 was picked because SLFN12 has been demonstrated to modulate CDH1 expression in prostate and breast cancers [12,13]. Although SLFN12 tended to change the mRNA levels of SCG1A1, SFTPC, HOPX, CDH1, CK-5, and P63, these changes were not comparable among all the tested cell lines. In addition, these observations did not seem to explain the clinical survival analysis. SGB1A1, SFTPC, and HOPX are all correlated with better survival in lung adenocarcinoma [32,33,34,35], while our results showed a reduction in mRNA of such markers in most of the cells. Both CK-5 and P63 are markers of lung squamous cell carcinoma [3]. Our mRNA data demonstrated no changes in p63, with a reduction of CK-5 in one cell line that models LUSC. Interestingly, SLFN12 significantly increased TTF-1 mRNA levels in two LUAD cell lines. Such results are consistent with the concept that SLFN12 is prognostically favorable in lung adenocarcinoma, because higher levels of TTF-1 are associated with favorable prognosis in non-squamous NSCLC [8]. Our mRNA data await further exploration at the protein level, particularly because SLFN12 can also regulate some proteins post-transcriptionally [11,12]. However, as the mRNA expression data did not seem to obviously explain the difference in the effect of SLFN12 on survival between LUAD and LUSC, we performed cell proliferation assays in the same cell models. Indeed, SLFN12 overexpression consistently reduced cell proliferation in LUAD cells without affecting LUSC cells, consistent with the human survival analysis. This antiproliferative effect of SLFN12 was further confirmed with Tag-it cell proliferation and tracking dye and flow cytometry analysis, which confirmed an actual proliferation reduction combined with cell cycle arrest with less cells entering mitotic phase, which further supports the reduction in proliferation induced by SLFN12 overexpression. Interestingly, SLFN12 overexpression increased the number of apoptotic cells in all of the three tested LUAD cell models. Although c-myc knockdown reported to induce apoptosis in lung cancer cells [36], it still needs to be determined if SLFN12 induces apoptosis through c-myc pathway or another independent pathway.

Gene expression analysis of two human lung cancer datasets revealed correlations of SLFN12 with different gene signatures. Importantly, the correlation of SLFN12 with the MYC, AKT-mTOR, MAPK, and beta-catenin gene signatures was different between LUAD and LUSC. Taking together the differential survival effect of SLFN12 in LUAD, but not in LUSC in two other databases, these results suggest that the role of SLFN12 in lung cancer may be specific to the histological subtype and not uniform across all lung cancers. Of these differentially correlated gene signatures, we chose c-myc for further study because of the differential correlation with the myc-downstream gene signature and the role of c-myc in cell cycle and proliferation [37].

C-myc is a transcriptional factor that is responsible for proliferation, differentiation, apoptosis, and maturation of cells through regulation of multiple genes [38,39,40,41]. C-myc has been established as an oncogene that is associated with poor prognosis and aggressive behavior in diverse cancers [41,42] and in particular promotes the proliferation of lung cancer cells [39,43]. Indeed, c-myc has been proposed as a therapeutic target for NSCLC or as a predicator for targeted chemotherapy [44,45]. Our proteomic analysis demonstrated a differential gene expression in LUAD versus LUSC, and further analysis showed that the correlation between SLFN12 and c-myc expressions was different in LUAD and LUSC. Therefore, we isolated c-myc for further investigation as a possible cause of different SLFN12 effect on survival and cell proliferation in LUAD versus LUSC. Overexpressing SLFN12 reduced the protein levels of c-myc in LUAD, but this was not the case in LUSC. This SLFN12 effect paralleled the results obtained from our survival analysis, cell proliferation study, and proteomic analysis.

The reduction in c-myc protein by SLFN12 contributes to its ability to inhibit cell proliferation, although SLFN12 may also regulate LUAD proliferation in other ways because c-myc protein co-expression attenuated rather than completely blocked the effect of SLFN12 on cell number. This suggests that alterations in c-myc are one of the axes through which SLFN12 exerts its action, while other downstream targets of SLFN12 await future exploration.

Although SLFN12 was previously reported to act through modulating proteasomal and USP14 and USCHL5 deubiquitylase activity [11], this did not appear to be the case in lung adenocarcinoma cells. Instead, SLFN12 acted through inhibiting the translation of c-myc protein, similar to how SLFN12 modulates the expression of ZEB1 in breast cancer [12]. This translational inhibition of c-myc by SLFN12 suggests a new potential pathway for future targeting of c-myc in lung adenocarcinoma.

Although we had observed that SLFN12 overexpression reduced c-myc, SLFN12 reduction, in contrast, did not significantly increase c-myc protein. This disparity might be explained in different ways. First, although SLFN12 was reduced in these experiments, it was not completely eliminated. Thus, the residual SLFN12 might have been sufficient to activate SLFN12-mediated pathways. Second, and conversely, basal SLFN12 levels in these cancer cells are already quite low. Thus, it is possible that overexpressing SLFN12 might have an effect, while there is simply not enough basal SLFN12 to have much of an effect on c-myc without or with further reduction by the shRNA. Third, SLFN12 appears to reduce c-myc at the translational, but not transcriptional levels. Thus, if c-myc is already being translated at a maximal rate at baseline in these cells, a reduction in SLFN12 might not cause a further increase in c-myc translation. Fourth, SLFN12 reduction surely affects proteins other than c-myc, which are likely to interact with c-myc transcription, translation, and degradation. Thus, tweaking the signaling web in different directions may have complex and not necessarily opposite effects. Distinguishing among these disparate and complex possibilities awaits further study beyond the scope of the current manuscript.

The question of how SLFN12 drives prognosis and why it is different in adenocarcinoma from squamous cell carcinoma awaits further exploration. Our results raise the possibility that SLFN12 may act in adenocarcinoma at least in part by its effects on c-myc and its downstream signals, but SLFN12 may affect a variety of transcription factors [11,12] and the interplay between them and their downstream effects is likely to be complex. Although adenocarcinoma of the lung and squamous cell carcinoma of the lung both derive from the same organ, their tumor biology and response to therapy are clearly different in many ways [46,47], and so one would not necessarily expect that the same stimulus would have the same effects in these very different tumor types.

Currently, there is no proven therapeutic drug that specifically targets SLFN12 expression, as until our current studies, there has been no particular thought that SLFN12 was important in cancer. This will clearly take time to develop. However, a recently patented specific PDE3A inhibitor compound has been identified (DNMDP) that appears to induce apoptosis in a set of NCI-60 cancer cells, including lung cancer cells, by enhancing the interaction of PDE3A with SLFN12. DNMDP worked only in cancer cells that exhibit high expression of SLFN12 [5]. This molecule might be useful to treat lung adenocarcinomas that express high levels of SLFN12, but this must await further study. However, even in the absence of a specific drug to target tumors on the basis of their SLFN12 levels, the ability to separate lung cancer patients into high versus low SLFN12 expression may help in predicting the prognosis and subsequent aggressiveness of the chemotherapy. On one hand, our data raise the possibility that lung adenocarcinomas with higher expression of SLFN12 might behave in a less aggressive fashion, and thus might be amenable to less aggressive cytotoxic regimen. On the other hand, future studies may develop drugs targeted at activating an SLFN12-mediated pathway that might be useful in lung adenocarcinomas that express low levels of SLFN12. Finally, because SLFN12 appears to affect many different aspects of cell biology, it remains possible that even more conventional chemotherapy may have different effects in tumors that express high or low SLFN12 levels. It is only by segregating patients into high and low SLFN12 expression groups that we will be able to discern differences in responsiveness in future chemotherapeutic studies.

Because direct targeting of c-myc in cancer is challenging, because of the pleiotropy of its transcription, indirect targeting of c-myc is a potential therapeutic approach [48]. SLFN12 and its downstream signals might offer another potential pathway to target c-myc in cancer by regulating its expression.

## 4. Materials and Methods

### 4.1. Survival Analysis

Survival analysis was performed using two different online publicly available tools. The first analysis was performed using the Kaplan Meier-plotter from www.kmplot.com, based on Affymetrix gene probes [14,15]. A total of 719 patients with LUAD and 524 patients with LUSC were divided into two groups based on the median expression values of SLFN12 (175 was the cutoff value for LUAD and 140 was a cutoff value of SLFN12 expression in LUSC) and the overall survival was compared between the two patient cohorts by a Kaplan–Meier survival plot, as well as the hazard ratio with 95% confidence intervals with the calculation of logrank *p*-value.

The second survival analysis was performed using Human Protein Atlas (http://www.proteinatlas.org) [16,17], which is based on RNA-Seq analysis, where 500 patients with LUAD and 494 patients with LUSC were grouped into two cohorts based on the best-cutoff (fragments per kilobase of exon model per million reads mapped) FPKM value of SLFN12 that gave the best separation of overall survival after diagnosis (2.25 and 1.57 were used as cutoff values of SLFN12 FPKM for LUAD and LUSC, respectively). Logrank *p*-value for the Kaplan–Meier plot was calculated.

### 4.2. Cells and Reagents

All cells were obtained from the American Tissue Culture Collection (ATCC). HCC827, H23, and H1975 cells were used as a model of lung adenocarcinoma (LUAD), while H2170 and HTB-182 cells were used to model lung squamous cell carcinoma (LUSC).

Cells were cultured in RPMI-1940 media (Genesee Scientific, El Cajon, CA, USA) supplemented with 10% fetal bovine serum (FBS) (Genesee Scientific) and 5% penicillin/streptomycin (ThermoFisher Scientific, Waltham, MA, USA) with 5% CO_2_ at 37 °C. MG132 and b-AP15 were obtained from selleckchem (Munich, Germany).

### 4.3. Viral Constructs

AdSLFN12 (Applied Biological Materials, Richmond, BC, Canada) was constructed using a pAdeno vector and the human SLFN12 insert (accession # NM_018042) combined with a CMV promoter. Control virus was constructed from the pAdeno vector with only the CMV promoter. Short hairpin RNA adenovector targeting SLFN12 was obtained from Vector Biolab (Malvern, PA, USA, #shADV-223642). C-Myc adenovirus was obtained from Vector Biolabs (Malvern, PA, USA, # 1285).

### 4.4. Viral Transfection

Seventy to eighty percent confluent cells in six-well plates were transduced at 2000–4000 viral particles/cell with the AdSLFN12 virus, AdC-MYC, Ad-ShRNA-SLFN12 or AdCMV, Ad-Sh-Scr as a control for twenty-four hours in 1 mL complete RPMI-1940 medium. After twenty-four hours, the culture medium was replaced with fresh RPMI medium and the cells were permitted to grow for 72–96 hours according to the study protocol.

### 4.5. Cell Viability

Cell viability was assayed using a water-soluble tetrazolium salt (Dojindo Molecular Technology, Rockville, MD, USA). In brief, cells were seeded at 5000 cells per well in 96-well plates in quadruplicates. Cells were allowed to adhere at 37 °C for 24 hours. Cells were then infected with adenoviral vectors at 2000–4000 viral particles per cell. Cell viability was serially measured at 0 and 96 hours after transfection in each plate. During measurements, 100 µL of fresh medium with 10% CCK8 solution was added per well and incubated at 37 °C for 2 hours, according to the manufacturer’s recommendations. Absorbance was measured with a BioTeck Epoch spectrophotometer (Winooski, VT, USA) at 450 nm.

Toluidine blue cell number assay was performed by seeding 25,000 cells per well in 24-well plates in triplicate. Cells were allowed to adhere at 37 °C for 24 hours and then infected with adenoviral vectors at 4000 viral particles per cell. Cell numbers were measured at 0 and 72 hours after infection. Cells were washed twice in phosphate buffered saline, fixed with ice-chilled methanol at −20 °C for 20 minutes, and stained with 1% toluidine blue for 45 minutes at room temperature. The cells were then washed with double distilled water and left to air dry for 2 hours. Cells were solubilized with 1% SDS solution and optical density was measured with the Spark^®^ Multimode Microplate Reader by Tecan (Männedorf, Switzerland) at 620 nm.

### 4.6. RNA and qPCR Studies

Total RNA isolation from cells was achieved using the RNeasy Mini Kit, Qiashredders, DNase treatment and the QiaCube instrument following the manufacturer’s protocols (Qiagen, Hilden, Germany). cDNA synthesis was performed using a SMARTScribe Reverse Transcription kit (Takara Clontech #639538, Mountain View, CA, USA) according to the manufacturer’s protocol. cDNA samples were analyzed by qPCR with BioRad CFX96 Touch Real-Time PCR Detection System and the PrimeTime Gene Expression Master Mix from Integrated DNA Technology (IDT, Coralville, IA, USA), according to the experimental design. Expression levels were determined from the threshold cycle (Ct) values using the method of 2-∆∆Ct using HPRT as the reference control genes. qPCR cycle conditions for Primer probe qPCR were 1 cycle of 2 minutes at 95 °C, and 50 cycles of 10 seconds at 95 °C and 45 seconds at the annealing temperature of 55 °C. Primers were obtained from Integrated DNA Technology (IDT). The set of primers/probe sequences used in this study was as follows:

HPRT; forward primer: TTGTTGTAGGATATGCCCTTGA, reverse GCGATGTCAATAGGACTCCAG, probe /5HEX/AGCCTAAGA/ZEN/TGAGAGTTCAAGTTGAGTTTGG/3IABkFQ/.

B2M; forward primer: GGACTGGTCTTTCTATCTCTTGT, reverse ACCTCCATGATGCTGCTTAC, probe /5HEX/CCTGCCGTG/ZEN/TGAACCATGTGACT/3IABkFQ/.

ACTB; forward primer: ACAGAGCCTCGCCTTTG, reverse CCTTGCACATGCCGGAG, probe /5HEX/TCATCCATG/ZEN/GTGAGCTGGCGG /3IABkFQ/.

POLR2A; forward primer: CAGTTCGGAGTCCTGAGTC, reverse TCGTCTCTGGGTATTTGATGC, probe /5HEX/ACTGAAGCG/ZEN/AATGTCTGTGACGGAG /3IABkFQ/.

TP63: Forward: CAAGAAACGAAGATCCCCAGA, reverse: GAGGAAGGTACTGCATGAGTTC, probe/5Cy5/TCACGGCCCCTCACTGGTAAGTAT/3IAbRQSp/.

SFTPC: Forward: GATGGTTCTGGAGATGAGCA, reverse: CTGGCTTGTAGGCGATCAG, probe /56-FAM/ATGGAGAAG/ZEN/GTGGCAGTGGTAACC/3IABkFQ/.

HOPX: Forward: GCAAACACAGCTTCCAAACC, reverse: CTCCGCTAGACCCTTCTCA, probe: /5Cy5/TGCCTCTTCCACACTAATGACAGACTG/3IAbRQSp/.

CDH1: Forward: GTCTGTCATGGAAGGTGCTC, reverse: CTGAGGATGGTGTAAGCGATG, probe: /56-FAM/AGACGCGGA/ZEN/CGATGATGTGAACAC/3IABkFQ/.

C-Myc: Forward: TCTTCCTCATCTTCTTGTTCCTC, reverse: TCCTCGGATTCTCTGCTCTC, probe: /5Cy5/TGGGCGGTGTCTCCTCATGG/3IAbRQSp/.

TTF-1: Forward: CAGACTTACTCCAAGCACCA, reverse: TTGGGAATGACTGGAAGACG, probe: /5Cy5/CTACTTCGGGCCACCATCTCACC/3IAbRQSp/. Ck-5 was obtained from BIO-RAD (cat#10031234) and the sequence is proprietary.

### 4.7. Western Blotting

Cells were seeded in six-well plates at 300,000 cell/well. On the next day, cells were infected with either AdSLFN12 or AdCMV as a control. After 24 hours, the culture medium was replaced with fresh RPMI medium supplemented with 10% FBS and 5% penicillin/streptomycin. Cells were allowed to grow for 48–72 hours after transfection and then lysed in Radioimmunoprecipitation assay (RIPA) lysis buffer supplemented with Halt protease inhibitors (ThermoFisher Scientific). Lysate protein was quantified using Bicinchoninic acid protein assay (ThermoFisher Scientific, #87786). Then, 30 µg of protein was loaded into 10% SDS-page gel electrophoresis, which was separated with 120 V, and then transferred into 0.2 µm nitrocellulose membranes using Tris-Glycine transfer buffer supplemented with 20% methanol at 4 °C for 18 hours with 32 V. Membranes were blocked with Odyssey blocking buffer (LI-COR, Lincoln, NE, USA) for one hour at room temperature followed by overnight incubation at 4 °C with the following primary antibodies: monoclonal anti-c-myc raised in rabbit (Cell Signaling Technology, Danvers, MA, USA, #5605), monoclonal anti-GAPDH raised in mouse (Meridian Life Science, Memphis, TN, USA), and monoclonal anti-Schlafen12 raised in rabbit (Abcam, Cambridge, MA, USA, #ab234418). Membranes were washed three times in Tris-buffered saline with 0.1% Tween (TBS-T) buffer for 5 minutes each and incubated for 1 hour at room temperature with appropriate secondary antibodies: Donkey anti-mouse CW800 and Donkey anti-rabbit RD680 (LI-COR) followed by three five-minute washes in TBS-T buffer. Membranes were dried for 1 hour and imaged using a LI-COR CLX machine. Data were analyzed using Image Studio v2.5 (LI-COR).

### 4.8. Click-iT Transation Assay

Cells seeded in 100 mm dish at 2.5 million cells/dish. On the next day, the cells were transfected with either AdSLFN12 or AdCMV as a control and incubated for 60 hours at 37 °C with 5% CO_2_. Cells were then washed twice, incubated with methionine-free RPMI for 1 hour, and then incubated with 50 µM L-AHA for 6 hours in methionine-free RPMI media supplemented with 10% FBS. Cells were lysed in lysis buffer (50 mM TrisHCL, 150 mM NaCl, 1% Triton X-100) and incubated with 500 µM EZ-Link Phosphine-PEG3-Biotin (ThermoFisher Scientific, # 88901) for 6 hours at 37 °C with mild agitation. C-myc was immunoprecipitated using a monoclonal antibody (Cell Signaling Technology, #5605) and magnetic beads (Bio-Rad, Hercules, CA, USA, #1614013) per the manufacturer’s protocol. The eluted protein was resolved using 10% SDS-PAGE and Western blot. Biotin was labeled with IRDye 800CW Streptavidin (LI-COR, #926–32230), and c-myc was labeled with IRDye 680LT Donkey anti-Rabbit IgG Secondary Antibodies. Images were acquired using a LI-COR-Clx, and analyzed using Image Studio (LI-COR).

### 4.9. Gene Expression and Signature Score Calculation

We retrieved raw data from two public databases, dataset 1 (*n* = 150; LUAD = 77, LUSC = 73) [19] and dataset 2 (*n* = 58; LUAD = 40, LUSC = 18) [20], collected from GEO or ArrayExpress and normalized using R Affy package. To determine the functionality of SLFN12, we computed the correlation between the gene-scores of SLFN12 with our previously published and functionally known gene signatures [18]. To compute gene signature scores, we applied the same method described previously in Ignatiadis M et al. [18] based upon the weighted average. Each module score was scaled within a study so that the 2.5% and 97.5% quantiles equaled +1 and −1, respectively. The entire analysis was performed using R/Bioconductor (www.r-project.org). Additionally, the correlation of SLFN12 with sets of specific genes was examined in both LUAD and LUSC.

### 4.10. Flow Cytometry

#### 4.10.1. Apoptosis

A total of 300,000 cells were seeded per well into six-well plates. On the following day, cells were infected with AdSLFN12 or AdCMV. After 72 hours, the cells were trypsinized and collected. Apoptotic cells were detected using Pacific Blue™ Annexin V Apoptosis Detection Kit with PI (BioLegend, #640928, San Diego, CA, USA) per the manufacturer’s protocol. Samples were acquired on a BD FACSymphony A3 flow cytometer (BD, San Jose, CA, USA) and analyzed with FlowJo software (TreeStar, Ashland, OR, USA).

#### 4.10.2. Cell Proliferation

Six million cells were incubated with 1 mL Phosphate Buffered Saline (PBS) that contained 5 µM Tag-IT violet dye (BioLegend, #425101) for 30 minutes at 37 °C, after which five milliliters of RPMI medium with 10% FBS was added to terminate the reaction, and the cells were centrifuged at 1200 rpm for 5 minutes. Cells were resuspended in fresh RPMI medium and seeded into six-well plates at a density of 300,000 cells per well. On the following day, the cells were serum-starved for six hours by incubation in serum-free RPMI medium, and then infected with AdSLFN12 or control AdCMV. Twenty-four hours after infection, the medium was replaced with fresh medium. Seventy-two hours after infection, the cells were trypsinized and collected in Falcon tubes (Corning, #352058, Glendale, AZ, USA) in 1000 µL fluorescence-activated cell sorting (FACS) buffer (PBS + 5% FBS). Samples were acquired on a BD FACSymphony A3 flow cytometer (BD) and analyzed with FlowJo software (TreeStar).

#### 4.10.3. Cell Cycle

A total of 300,000 cells were seeded per well into six-well plates. On the following day, the cells were starved by incubation in serum-free RPMI medium for 6 hours. The cells were then infected with AdSLFN12 or AdCMV. After 72 hours, the cells were trypsinized, and incubated with 5 µM Vybrant dye cycler green (Thermo Fisher Scientific, #V35003) for 30 minutes at 37 °C in the dark. Samples were acquired on a BD FACSymphony A3 flow cytometer (BD) and analyzed with FlowJo software (TreeStar).

## 5. Conclusions

Our results indicate that SLFN12 does play a role in lung cancer biology. In particular, SLFN12 seems important for LUAD, but not LUSC. This may be important both for prognostic assessment and for future precision-targeted therapy in lung adenocarcinoma.

## Supplementary Materials

The following are available online at https://www.mdpi.com/2072-6694/12/10/2738/s1, Figure S1: Detailed information about western blot, Figure S2: Validation of HPRT stability as a housekeeping gene, Figure S3: SLFN12 effect on differentiation markers in lung adenocarcinoma cells, Figure S4: Schlafen 12 induces apoptosis in lung adenocarcinoma cells, Figure S5: Schlafen 12 slows the proliferation of lung adenocarcinoma cells, Figure S6: Schlafen 12 induces cell cycle arrest in lung adenocarcinoma cells, Figure S7: Schlafen 12 correlation with lung adenocarcinoma markers, Figure S8: SLFN12 reduction does not alter C-myc protein levels, Table S1: LUAD, Table S2: LUSC, Table S3: LUSA vs. LUSC.
