# Supplementary material for: Schlafen 12 Is Prognostically Favorable and Reduces C-Myc and Proliferation in Lung Adenocarcinoma but Not in Lung Squamous Cell Carcinoma
Source: Cancers (Basel). 2020 Sep 24;12(10):2738. doi: 10.3390/cancers12102738 (PMC7650563; doi:10.3390/cancers12102738)
Supplement: Supplementary file 1 [file cancers-12-02738-s001.zip › cancers-943216 - final - supplementary material/cancers-943216 - supplementary material.docx]

Supplementary Materials

Schlafen 12 is Prognostically Favorable and Reduces C-Myc and Proliferation in Lung Adenocarcinoma but Not in Lung Squamous Cell Carcinoma

Sarmad Al-Marsoummi, Jonathan Pacella, Kaylee Dockter, Matthew Soderberg,
Sandeep K. Singhal, Emilie E. Vomhof-DeKrey and Marc D. Basson


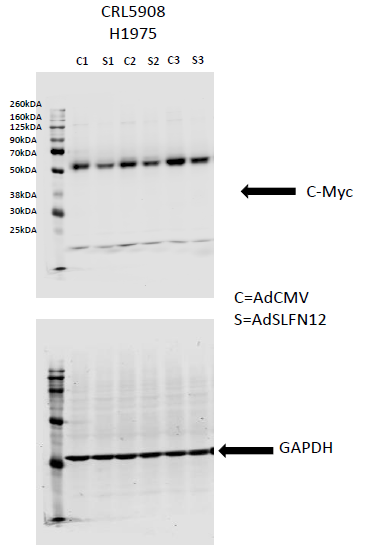


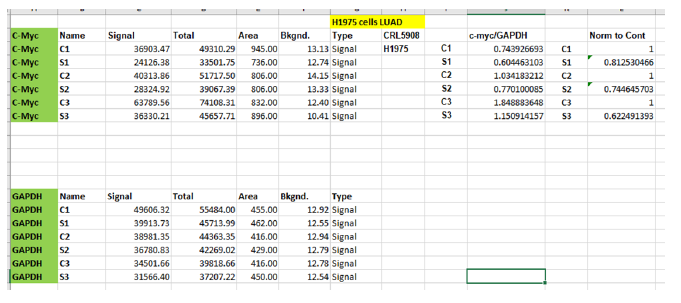


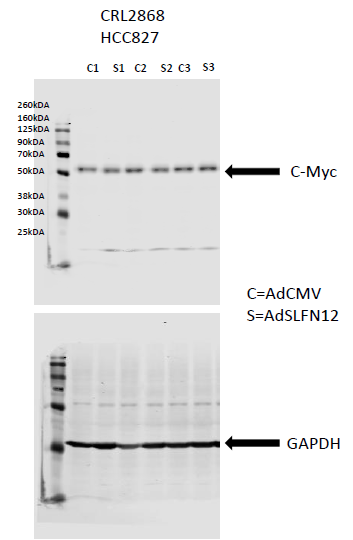


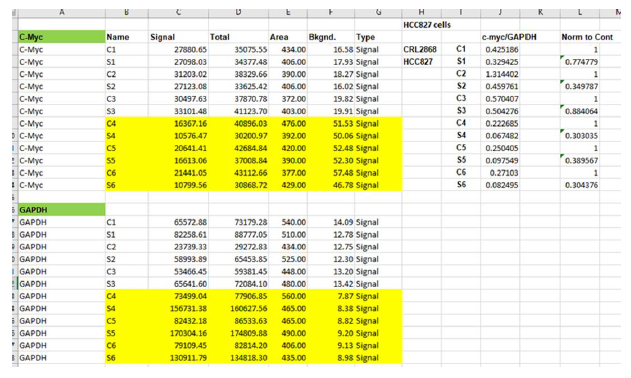


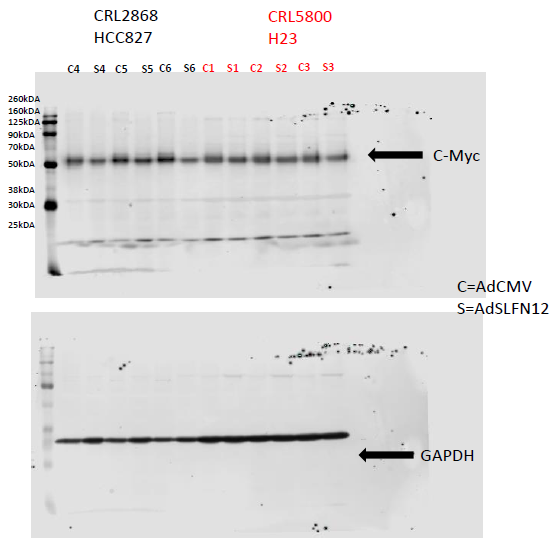


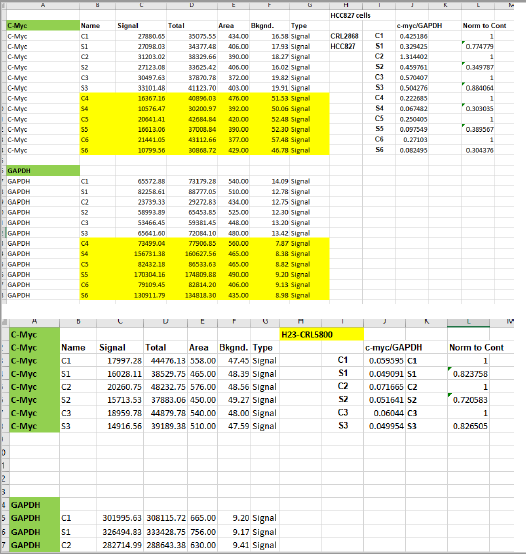


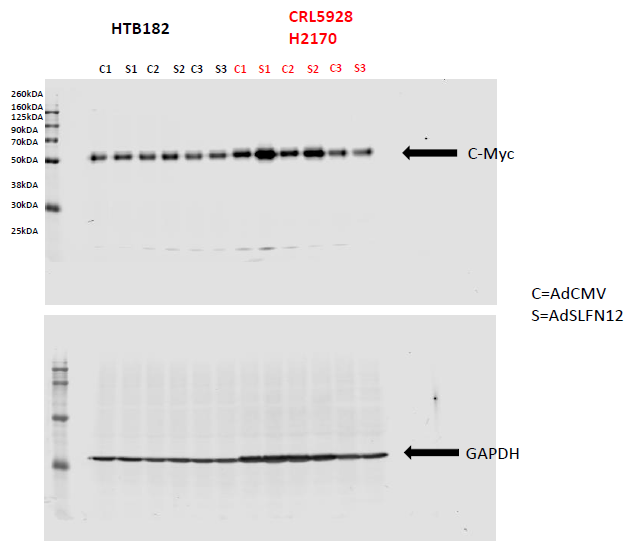


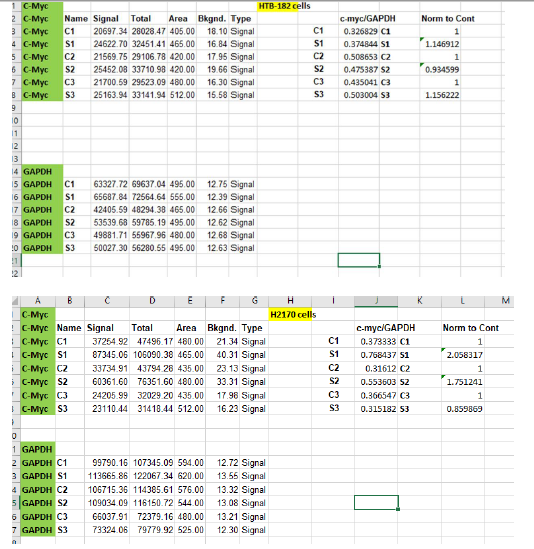


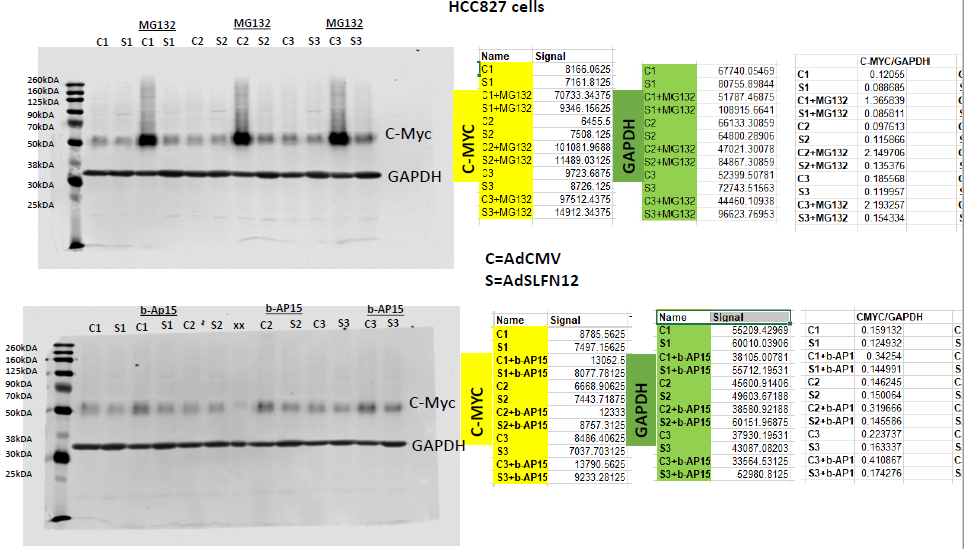


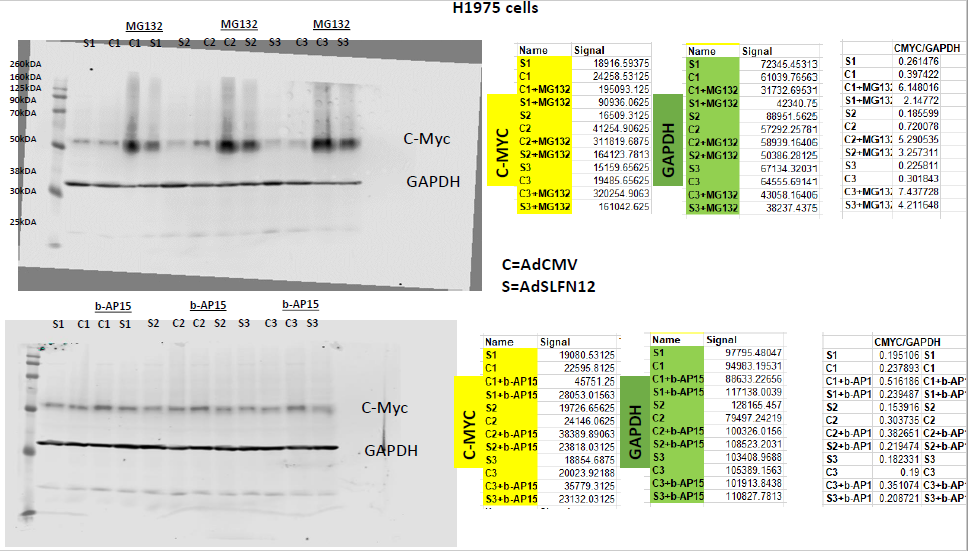


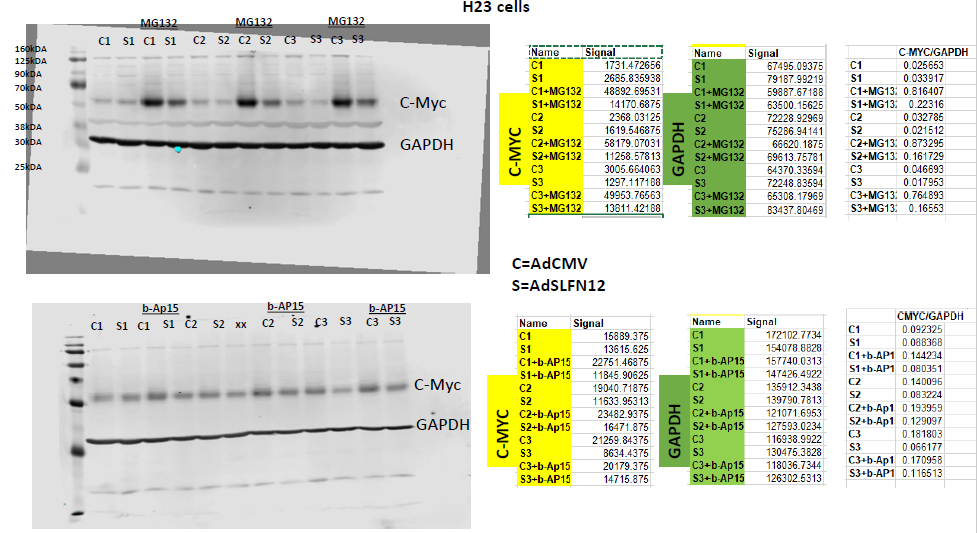


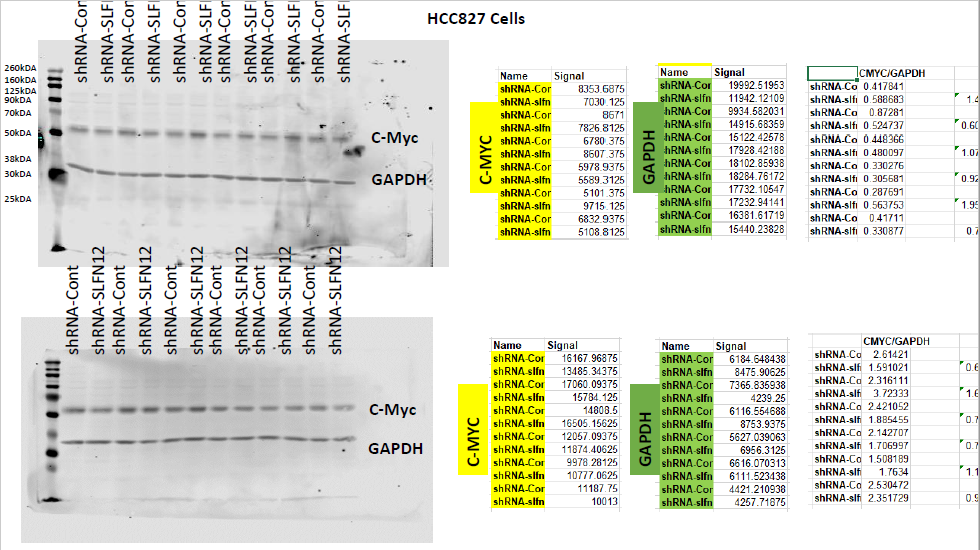


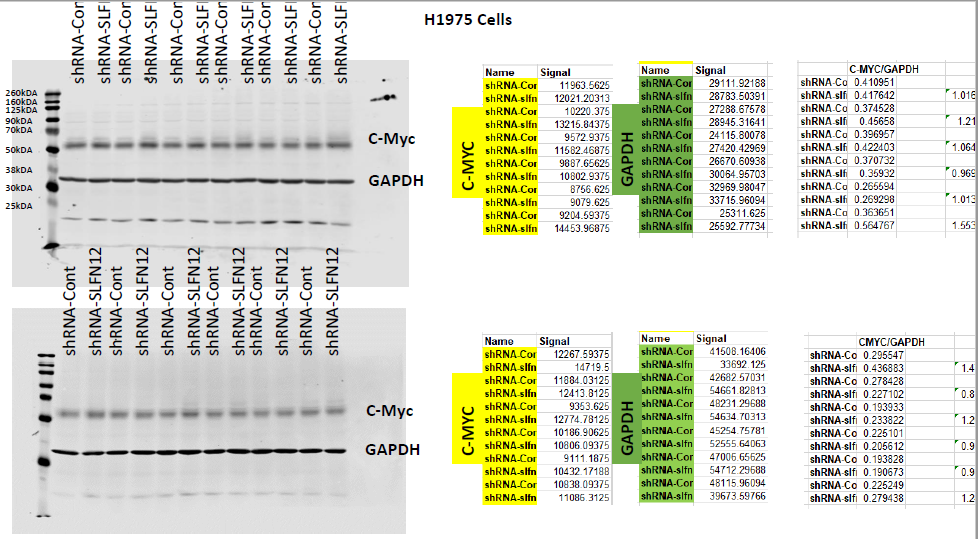


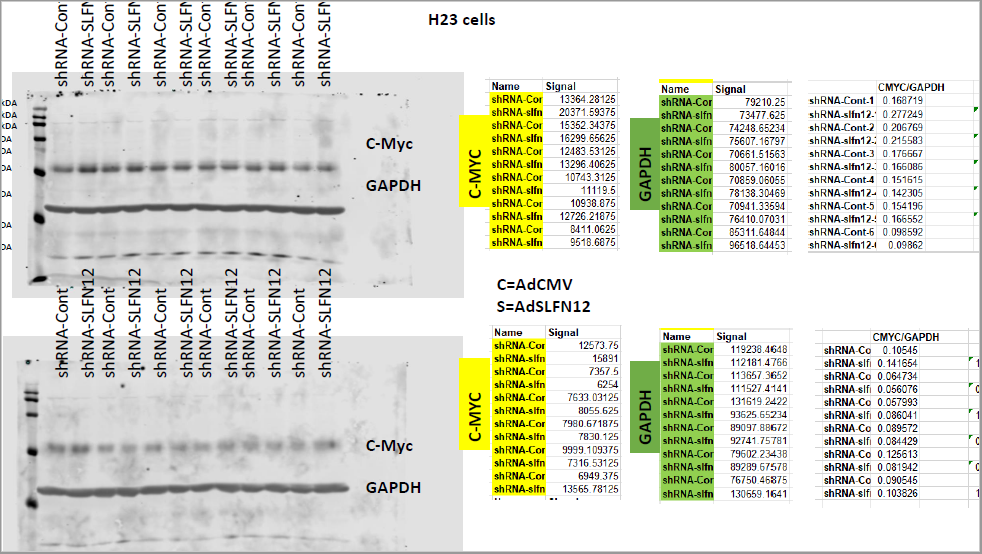


**Figure S1.** Detailed information about western blot.


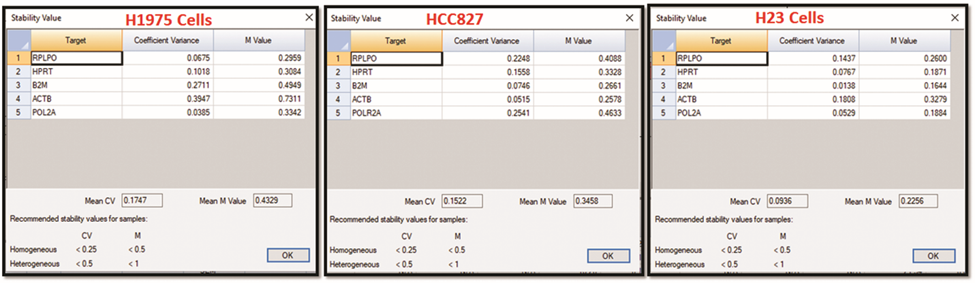


**Figure S2.** Validation of HPRT stability as a housekeeping gene. RT-qPCR primer-probe analysis of mRNA levels of multiple housekeeping genes in H1975, HCC827, and H23 lung adenocarcinoma cells with subsequent stability analysis using Bio-Rad CFX manager v.3.1 shows that the coefficient of variation (CV) and the average expression stability (M) values of HPRT are within the recommended values of a stable housekeeping gene expression, validating HPRT as a stable housekeeping gene for the purposes of these experiments.





**Figure S3.** SLFN12 effect on differentiation markers in lung adenocarcinoma cells. mRNA-level analysis by primer-probe RT-qPCR of SFTPC, SCGB1A1, HOPX, P63, CK-5, and CDH1 in H1975, HCC827, and H23 lung adenocarcinoma cells at 48 and 72 hours after transfection with eitherAdSLFN12 or AdCMV control shows a complex and inconsistent pattern. HPRT was used as a reference gene (* *p* < 0.05). All data are represented as mean ± SEM.


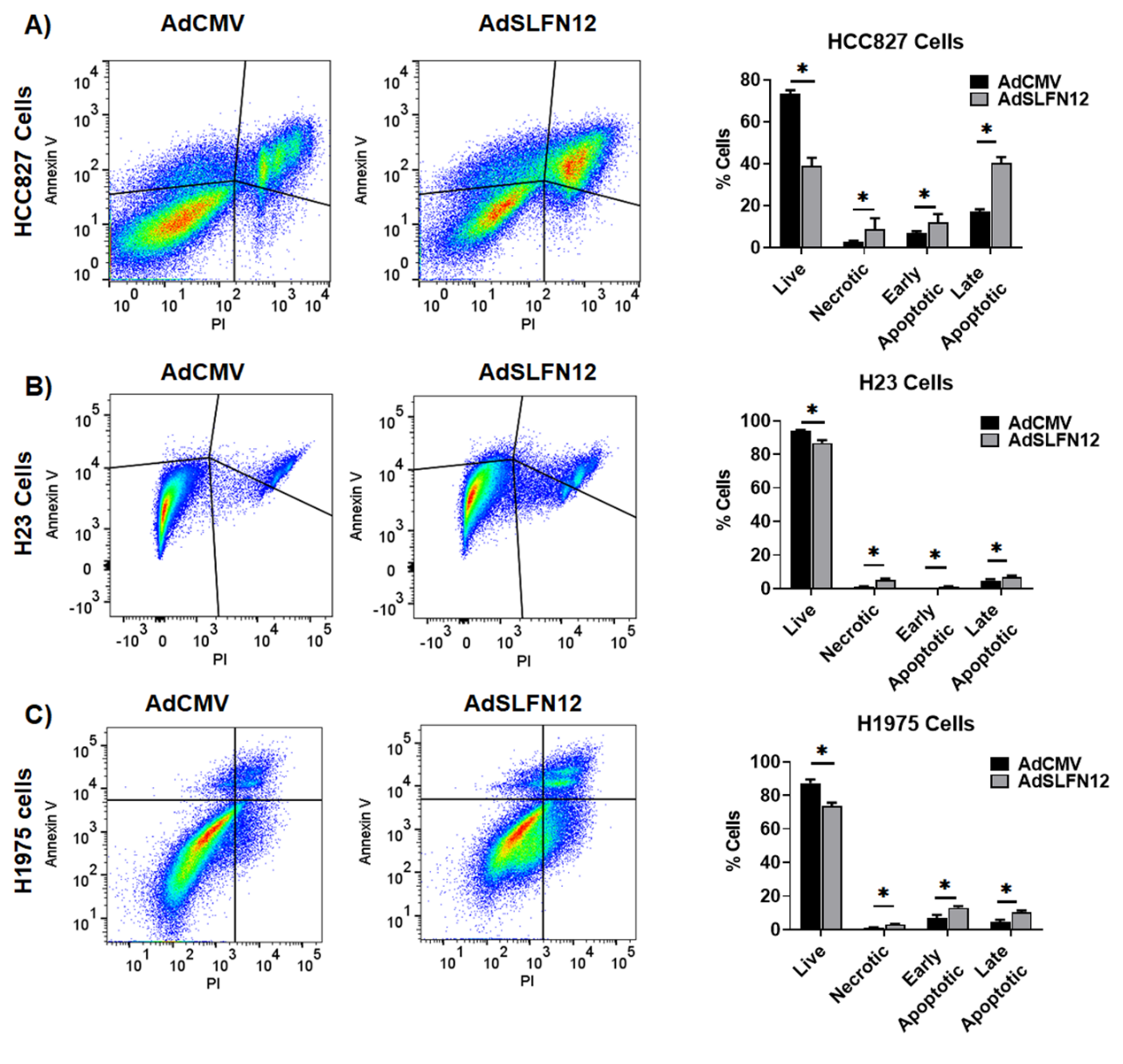


**Figure S4.** Schlafen 12 induces apoptosis in lung adenocarcinoma cells. Flow cytometric analysis of apoptotic cells using Annexin V/Propidium iodide labeling 72 hours after SLFN12 overexpression AdSLFN12 in: **(A)** HCC287 cells (*n* = 4) **(B)** H23 cells (*n* = 4), and **(C)** H1975 cells (*n* = 5). There was an increase in the proportion of apoptotic cells among cells infected with AdSLFN12 compared with cells infected with AdCMV control. (* *p* < 0.05). All data are represented as mean ± SEM.


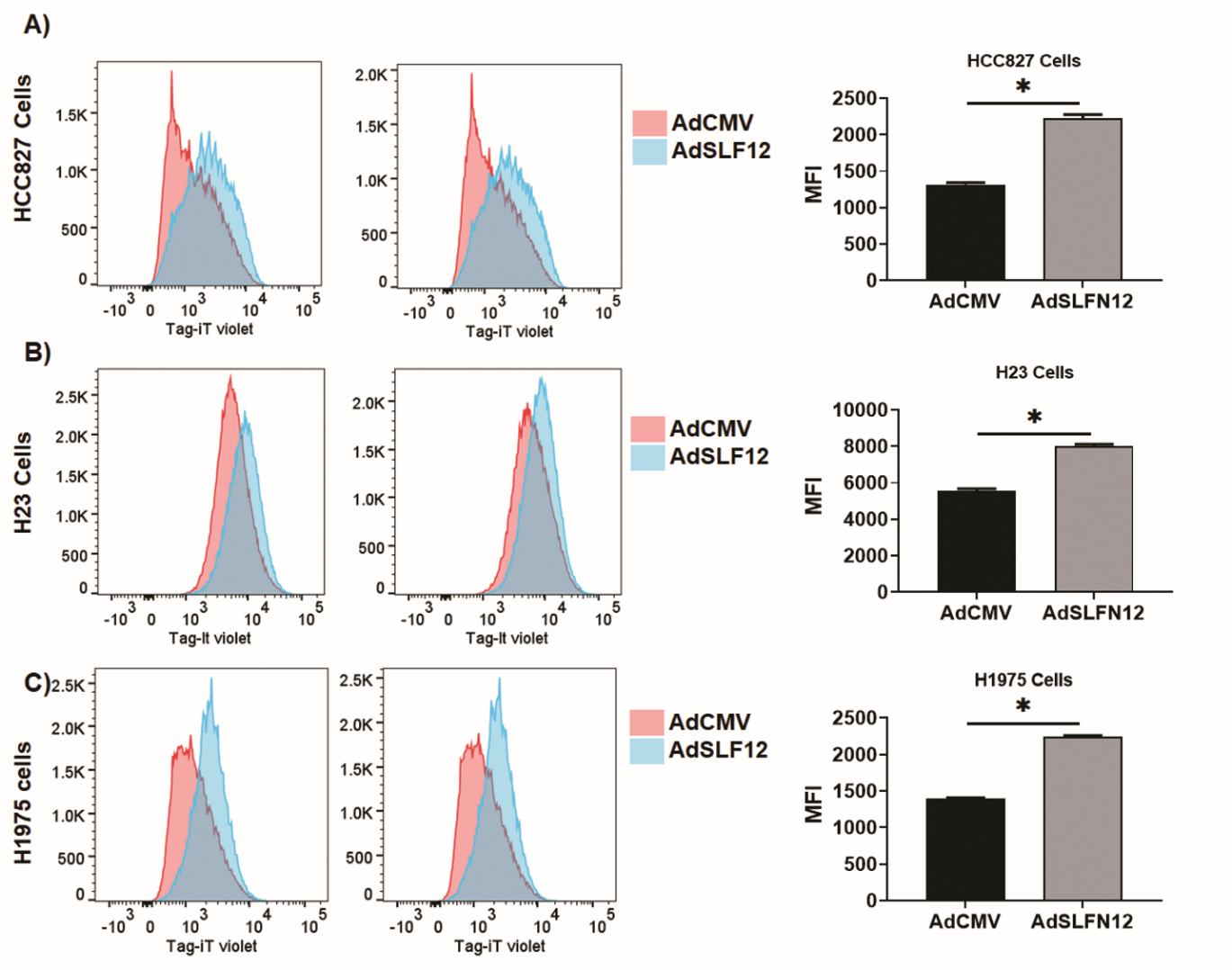


**Figure S5.** Schlafen 12 slows the proliferation of lung adenocarcinoma cells. Flow cytometric analysis of cell proliferation using Tag-it Violet labeling of cells 72 hours after SLFN12 overexpression using AdSLFN12 in: **(A)** HCC287 cells (*n* = 6), **(B)** H23 cells (*n* = 3), and **(C)** H1975 cells (*n* = 3). There was a significant reduction in cell proliferation in AdSLFN12-infected cells compared to the cells infected with AdCMV control. Mean fluorescence intensity (MFI) indicates more retention of Tag-it dye within the cells, which correlates with less proliferation (* *p* < 0.05). All data are represented as mean ± SEM.





**Figure S6.** Schlafen 12 induces cell cycle arrest in lung adenocarcinoma cells. Flow cytometric analysis of cell cycle using Vybrant dyecycle green labeling of the cells, 72 hours after SLFN12 overexpression using AdSLFN12 in: **(A)** HCC287 cells (*n* = 5), **(B)** H23 cells (*n* = 6), and **(C)** H1975 cells (*n* = 3). Cells were synchronized by serum starvation for six hours prior to infection. There was a significant increase in the proportion of cells arrested in G0/G1 phase, with significantly less cells entering M-phase of the cell cycle. Cells infected with AdCMV were used as a control. (* *p* < 0.05). All data are represented as mean ± SEM.


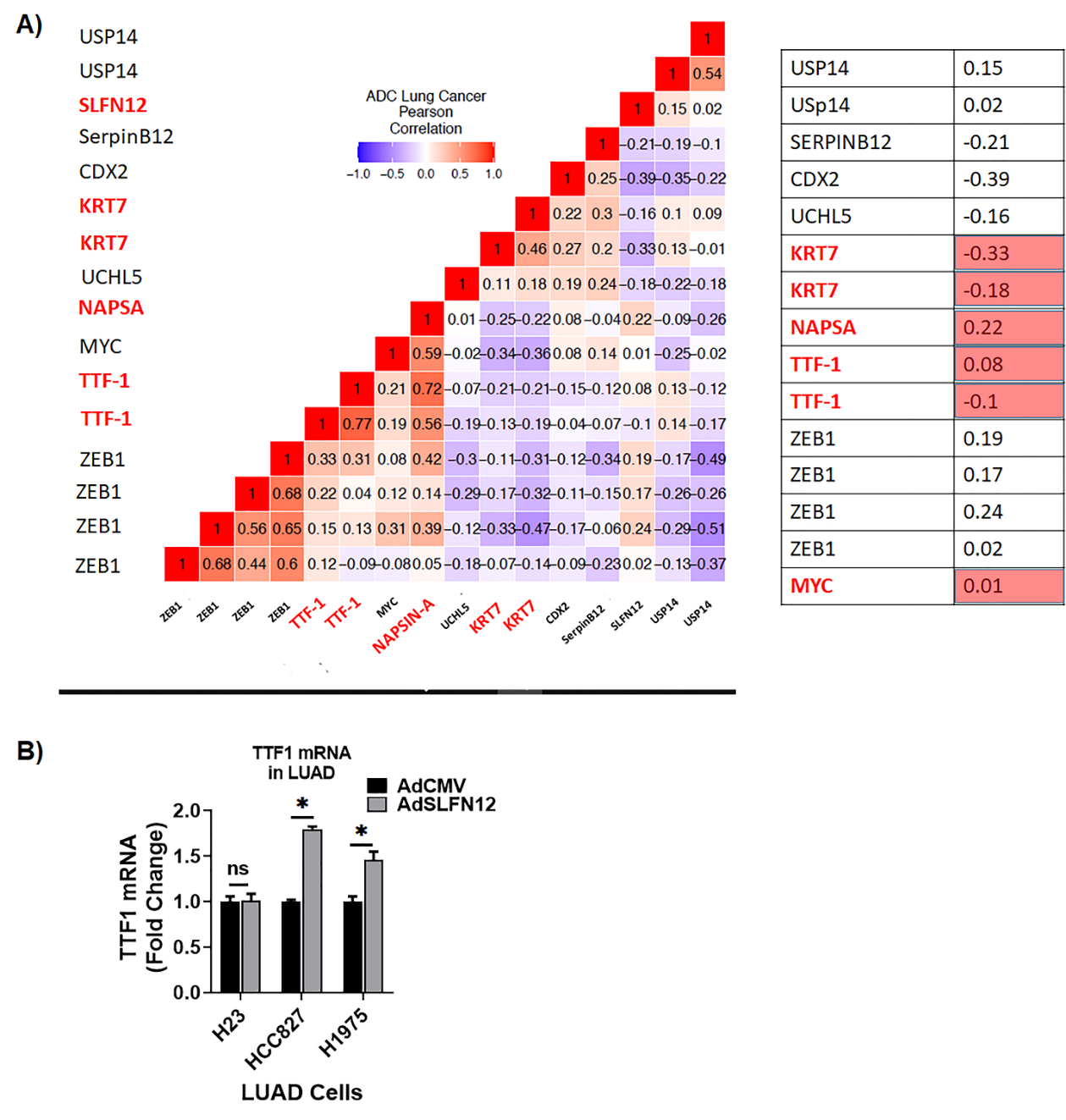


**Figure S7.** Schlafen 12 correlation with lung adenocarcinoma markers. (**A**) Bioinformatics analysis of a datasets of 117 lung adenocarcinomas shows the correlation of SLFN12 with the lung adenocarcinoma markers KRT7, NAPSA, and TTF1 in that dataset. (**B**) TTF-1 mRNA analysis by primer-probe RT-qPCR 72 hours after SLFN12 overexpression using AdSLFN12 in lung adenocarcinoma cells: H23 (*n* = 3), HCC827 (*n* = 3), and H1975 cells (*n* = 3). AdSLFN12 significantly increased TTF-1 mRNA levels in HCC827, and H1975 with no changes in H23 cells (AdCMV used as a control, HPRT used as a reference gene, * *p* < 0.05).


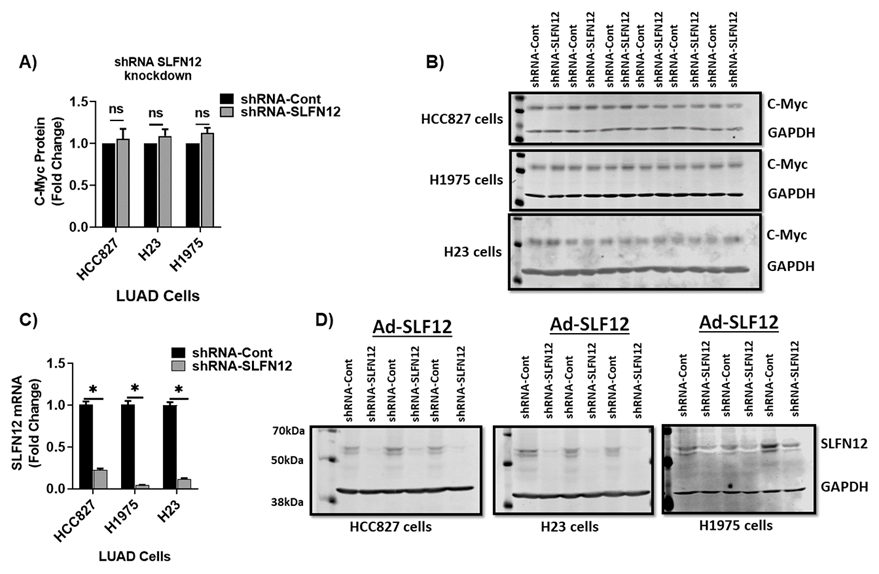


**Figure S8.** SLFN12 reduction does not alter C-myc protein levels. **(A)** C-myc protein levels analyzed by western blot 72 hours after reduction of Schlafen 12 using adenoviral vector expressing short hairpin RNA targeting SLFN12 (shRNA-SLFN12) in lung adenocarcinoma cells: HCC827 (*n* = 12), H23 (*n* = 12), and H1975 cells (*n* = 12). Representative western blot images are shown in **(B).** Adenoviral vector expressing non-targeted small hairpin RNA (shRNA-Cont) was used as a control and GAPDH served as a reference protein. (ns = non-significant). **(C)** SLFN12 mRNA-level analysis by primer-probe RT-qPCR 72 hours after sh-SLFN12 or shRNA-Cont treatment in HCC827 cells (*n* = 3), H1975 cells (*n* = 3), and H23 cells (*n* = 3). HPRT was used as a reference gene. (* *p* < 0.05). **(D)** Because endogenous SLFN12 protein levels in these cells are relatively low and difficult to detect by conventional western blot, we confirmed our RT-qPCR data showing that message level reduction of endogenous SLFN12 by the shRNA seen in **(C)** by adenovirally overexpressing SLFN12 in the presence of the SLFN12 directed shRNA (shRNA-SLFN12) or control shRNA (shRNA-Cont). These experiments confirmed that the shRNA directed against SLFN12 was able to substantially reduces even virally overdriven SLFN12 protein levels. All data are represented as mean ± SEM. Detailed information about western blot can be found at Figure S8.

| 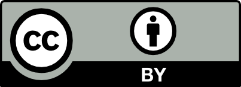 | © 2020 by the authors. Licensee MDPI, Basel, Switzerland. This article is an open access article distributed under the terms and conditions of the Creative Commons Attribution (CC BY) license (http://creativecommons.org/licenses/by/4.0/). |
| --- | --- |
